# FOXC1 promotes melanoma by activating MST1R/PI3K/AKT pathway and is associated with poor prognosis in melanoma

**DOI:** 10.18632/oncotarget.11224

**Published:** 2016-08-11

**Authors:** Jinhua Wang, Li Li, Shiwei Liu, Ying Zhao, Lin Wang, Guanhua Du

**Affiliations:** ^1^ The State Key Laboratory of Bioactive Substance and Function of Natural Medicines, Beijing Key Laboratory of Drug Target Research and Drug Screen, Institute of Materia Medica, Chinese Academy of Medical Science and Peking Union Medical College, Beijing 100050, China; ^2^ Department of Endocrinology, Shanxi DAYI Hospital, Shanxi Medical University, Taiyuan, Shanxi 030002, China; ^3^ Department of Molecular Oncology, John Wayne Cancer Institute (JWCI) at Providence Saint John's Health Center, Santa Monica 90404, CA, USA

**Keywords:** FOXC1, cutaneous melanoma, MST1R/PI3K/AKT pathway, methylation

## Abstract

FOXC1 is a member of Forkhead box family transcription factors. We showed that FOXC1 level was increased in melanoma cells and tissues and correlated with hypomethylation of the FOXC1 gene. Overexpression of FOXC1 promoted proliferation, migration, invasion, colony formation and growth in 3D Matrigel of melanoma cells. FOXC1 increased MST1R and activated the PI3K/AKT pathway. Also, FOXC1 expression was associated with disease progression and poor prognosis of melanoma. We suggest that FOXC1 is a potential prognostic biomarker for treating melanoma and predicting outcome of patients.

## INTRODUCTION

Melanoma, a malignancy originating in pigment-producing melanocytes, is the most aggressive form of skin cancer, and its incidence has been increasing worldwide [1]. Melanoma cells have a high tendency to metastasize the brain and liver [2], and five-year survival rates for patients with distant metastatic disease remain below 20% [3]. Moreover, it remains difficult to predict melanoma recurrence after surgical resection of early-stage metastatic melanomas.

Forkhead box (Fox) proteins are a family of evolutionarily conserved transcriptional regulators defined by a common DNA-binding domain (DBD) termed the forkhead box or winged helix domain [4]. Fox protein family members play important roles in both healthy biological processes and cancer development, affecting metabolism, development, differentiation, proliferation, apoptosis, migration, invasion and longevity [5]. FOXM1 regulates expression of eukaryotic elongation factor 2 kinase and promotes proliferation, invasion and tumorgenesis of human triple negative breast cancer cells [6]. FOXM1 is also associated with high-risk multiple myeloma [7] and ovarian cancer [8]. FOXF1 was associated with the development of esophageal adenocarcinoma [9] and promotes migration of breast cancer cells by upregulating lysyl oxidase and suppressing Smad2/3 signaling [10]. FOXQ1 mediates the crosstalk between TGF-beta and Wnt signaling pathways in the progression of colorectal cancer [11] and is associated with poor prognosis of pancreatic cancer [12]. In contrast, FOXA1 and FOXA2 are known to inhibit the metastasis of pancreatic ductal adenocarcinoma and lung cancer through the transactivation of E-cadherin expression and maintenance of the epithelial phenotype [13]. FOXP3 was found to inhibit melanoma tumorigenesis via effects on proliferation and apoptosis [14]. In addition, FOXC1 was recently shown to be involved in the development of some cancers. Our earlier studies showed that FOXC1 is a potential biomarker for basal-like breast cancer (BLBC), and its overexpression correlates with poor overall survival in breast cancer [15]. Overexpression of FOXC1 also promotes tumor metastasis and indicates a poor prognosis in hepatocellular carcinoma [16], is associated with poor clinical outcome in non-small cell lung cancer patients and with a poor prognosis in pancreatic ductal adenocarcinoma [17, 18], correlates with poor prognosis in gastric cancer patients [19]. However, there is no report on the clinicopathologic significance of FOXC1 in melanoma, and the role played by FOXC1 in melanoma has not yet been determined.

In this study, we investigated the expression and function of FOXC1 in melanoma. Our findings indicate FOXC1 acts via the MSTR1/PI3K/AKT pathway, and its expression is related to disease progression and predicts a poor prognosis in melanoma patients. They also suggest FOXC1 could potentially be served as a useful therapeutic target in melanoma.

## RESULTS

### FOXC1 is highly expressed in melanoma

**Figure 1:**
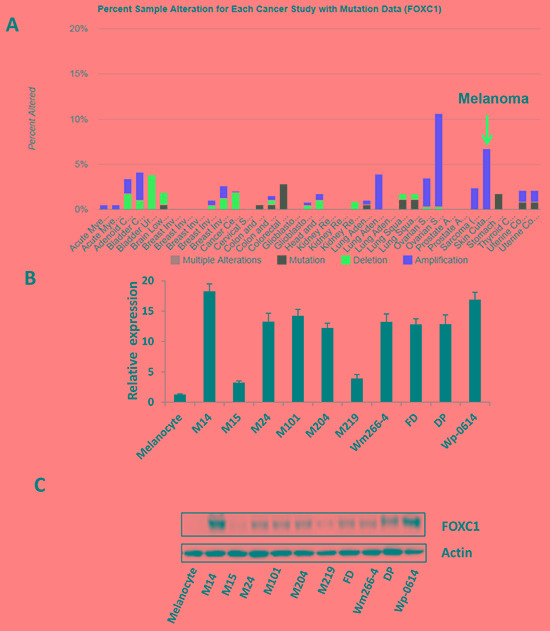
FOXC1 expression in melanoma **A.** Alteration of FOXC1 expression (6-7%) was amplified in skin cutaneous melanoma *(N=319)* of *TCGA* (The Cancer Genome Atlas). **B.** There is higher FOXC1 mRNA expression in melanoma cell lines (N=11) than that in melanocyte by qRT-PCR. **C.** There is higher FOXC1 protein expression in melanoma cell lines (N=11) than that in melanocyte by western blot.

To assess the FOXC1 expression in melanoma, silicon assay was performed using TCGA database (http://www.cbioportal.org/public-portal/cross_cancer.do). Results showed that alternation of FOXC1 was amplified in melanoma (Figure 1A). We also explored relation between FOXC1 mRNA and amplification, the result was shown in Supplementary Figure S1. To check FOXC1 expression in melanoma cell lines, real time PCR and western blot were performed. It was shown in Figure 1B and 1C there was high FOXC1 expression (mRNA and protein) in most of melanoma cell lines.

### FOXC1 promotes proliferation and enhances migration and invasion of melanoma cell

**Figure 2:**
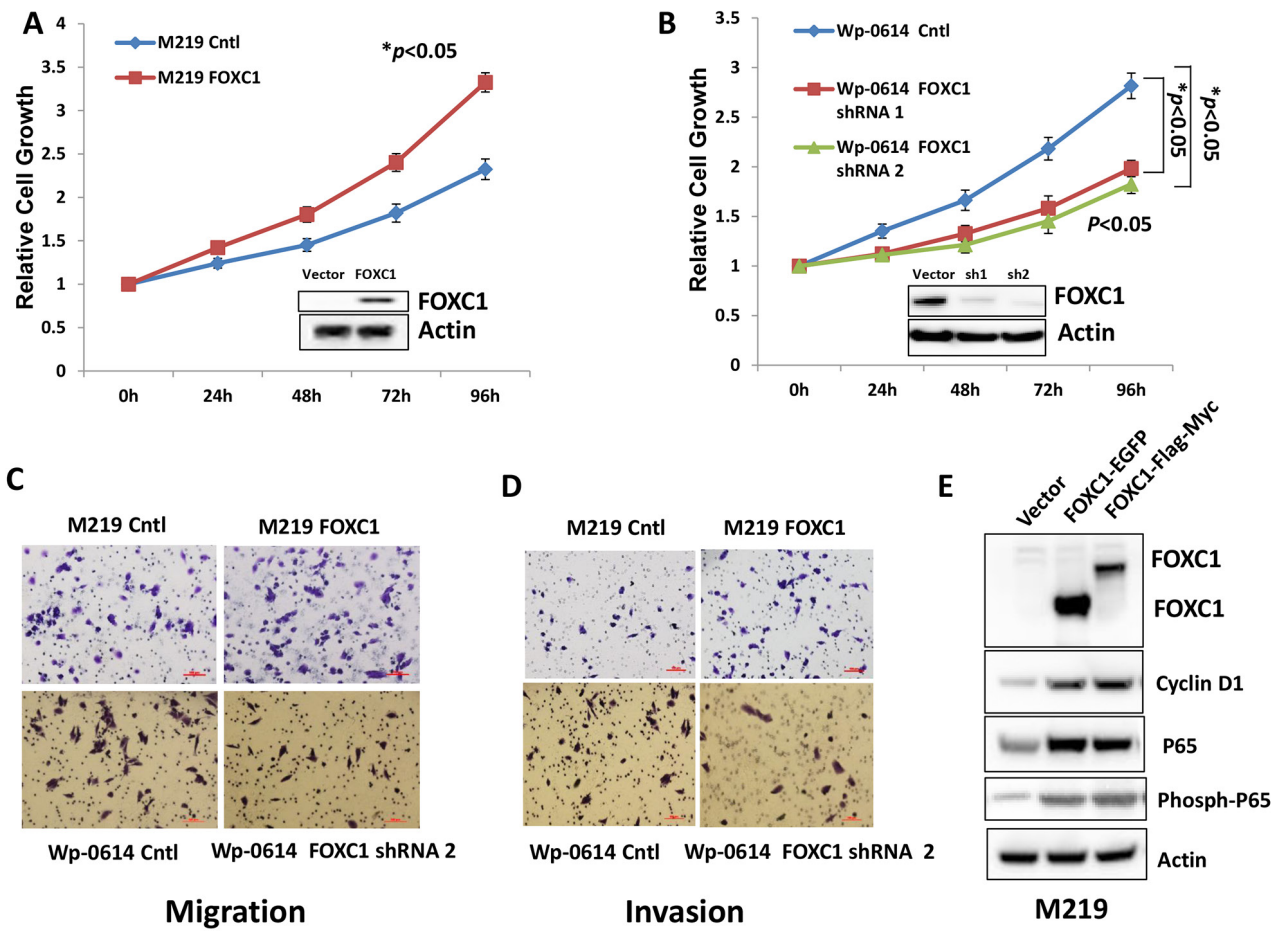
FOXC1 promotes proliferation, migration and invasion of melanoma cells **A.** The growth of M219 FOXC1 is higher than that of M219 control. **B.** The growth of Wp-0614 FOXC1 shRNA is lower than that of Wp-0614 control. **C.** FOXC1 overexpression increased cell migration while knockdown of FOXC1 reduced cell migration. **D.** FOXC1 overexpression increased cell invasion while knockdown of FOXC1 reduced cell invasion. **E.** FOXC1 overexpression induced expression of Cyclin D1, P65 and phosph-P65, which are related to growth, migration and invasion of melanoma cells. Error bars, s.d. (*p <0.05).

To explore the function of FOXC1 in melanoma cell lines, we transfected M219 cells which have low FOXC1 expression with FOXC1-myc-flag and Wp-0614 cells which have high FOXC1 expression with FOXC1 shRNAs. M219 cell clones which have high FOXC1 and Wp-0614 cell clones which have low FOXC1 were selected by G418 and puromycin, respectively. Cell growth, migration and invasion were compared between M219 control, M219 FOXC1 and Wp-0614 control, Wp-0614 FOXC1 shRNA 1, Wp-0614 FOXC1 shRNA2. It was shown in Figure 2A and 2B that growth of M219 FOXC1 was higher than that of M219 control while growth of Wp-0614 FOXC1 shRNAs was slower than that of Wp-0614 control. Results also showed that migration and invasion of M219 FOXC1 was higher than that of M219 control while migration and invasion of Wp-0614 FOXC1 shRNA2 was lower than that of Wp-0614 control (Figure 2C and 2D). Migration and invasion of Wp-0614 FOXC1 shRNA1 were similar to Wp-0614 FOXC1 shRNA 2 (Data wasn't shown). Quantification of cell migration and invasion was shown in Supplementary Figure S2A and S2B. Cyclin D1 and P65 are related with growth, migration and invasion. Our previous studies showed FOXC1 increases expression of Cyclin D1, phosph-P65 and P65 in breast cancer cells. Immunoblot analysis further confirmed the results in melanoma (Figure 2E). All in all, these results supported that FOXC1 promotes aggressive character of melanoma cells.

### FOXC1 increases colony formation and growth of 3D matrigel of melanoma cells

**Figure 3:**
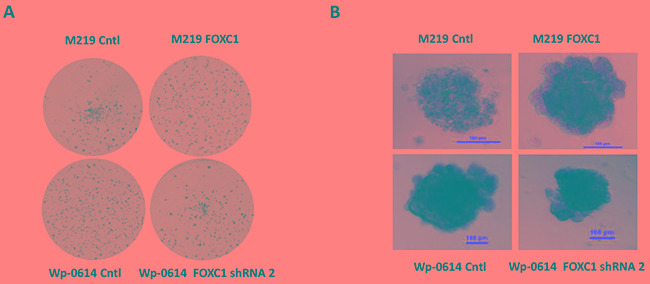
FOXC1 promotes colony formation and growth in 3D matrigel **A.** FOXC1 increased colony formation of melanoma cells in Soft Agar while knockdown of FOXC1 reduced colony formation of melanoma cells. **B.** Growth of M219 FOXC1 in 3D matrigel is higher than that of M219 control in 3D matrigel while growth of Wp-0614 FOXC1 shRNA in 3D matrigel is lower than that of Wp-0614 in 3D matrigel.

To assess if FOXC1 promotes tumorigenesis of melanoma, we performed colony formation assay in Soft Agar and 3D matrigel culture. Results showed that M219 FOXC1 cells grew faster than M219 control in Soft Agar and 3D matrigel culture while growth of Wp-0614 FOXC1 shRNA2 was lower than that of Wp-0614 control in Soft Agar and 3D matrigel culture (Figure 3A and 3B). Growth of Wp-0614 FOXC1 shRNA1 in Soft Agar and 3D matrigel culture was similar to that of Wp-0614 FOXC1 shRNA 2 (Data wasn't shown).

### High FOXC1 expression is due to hypomethylation of FOXC1 gene

**Figure 4:**
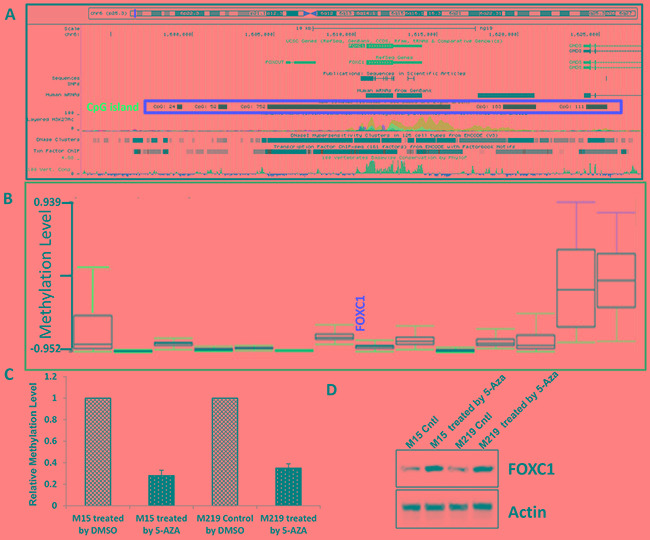
High FOXC1 expression was associated with hypomethylation of FOXC1 gene **A.** There is a big 752bp CpG island in the promoter region of FOXC1 gene. **B.** Data from TCGA skin cutaneous melanoma (N=319) showed that methylation level of FOXC1 gene was very low. **C.** Methylation level of FOXC1 promoter in M15 control and M15 treated by 5-Aaz, M219 control and M219 treated by5-Aza was shown. Methylation level of FOXC1 promoter was reduced when cells were treated by 5-Aza. **D.** The expression of FOXC1 in melanoma cell lines with low FOXC1 expression was induced by treatment with 5-Aza for 72 hours.

Previous study showed that the expression of FOXC1, one of the transcription factors hypomethylated and highly expressed in CD^44+^ cells, induced a progenitor-like phenotype in differentiated mammary epithelial cells. To explore the methylation level of FOXC1 gene in melanoma, silicon assay was done using UCSC gene browser (http://genome.ucsc.edu) and TCGA data from Cancer Genomics Browse (https://genome cancer.ucsc.edu/proj/site/hgHeatmap/). It was shown that there are rich CpG islands (752bp) in the promoter region of FOXC1 gene (Figure 4A) and that there was very low methylation level of FOXC1 gene in TCGA melanoma (Figure 4B). To further investigate whether methylation of FOXC1 gene is related to expression, M219 and M15 cells which have low FOXC1 expression were treated with a 5-Aza demethylation agent and expression of FOXC1 protein was assessed for by immunoblotting. Results showed that methylation of FOXC1 gene was closely correlated with FOXC1 expression (Figure 4C). Western blot further confirmed that FOXC1 expression in M219 and M15 was increased when methylation was reduced (Figure 4D). Taken together, these results suggested that there was hypomethation of *foxc1* and FOXC1 expression was closely associated with methylayion of *foxc1 in melanoma*.

### FOXC1 promotes melanoma cell function by regulating MST1R

**Figure 5:**
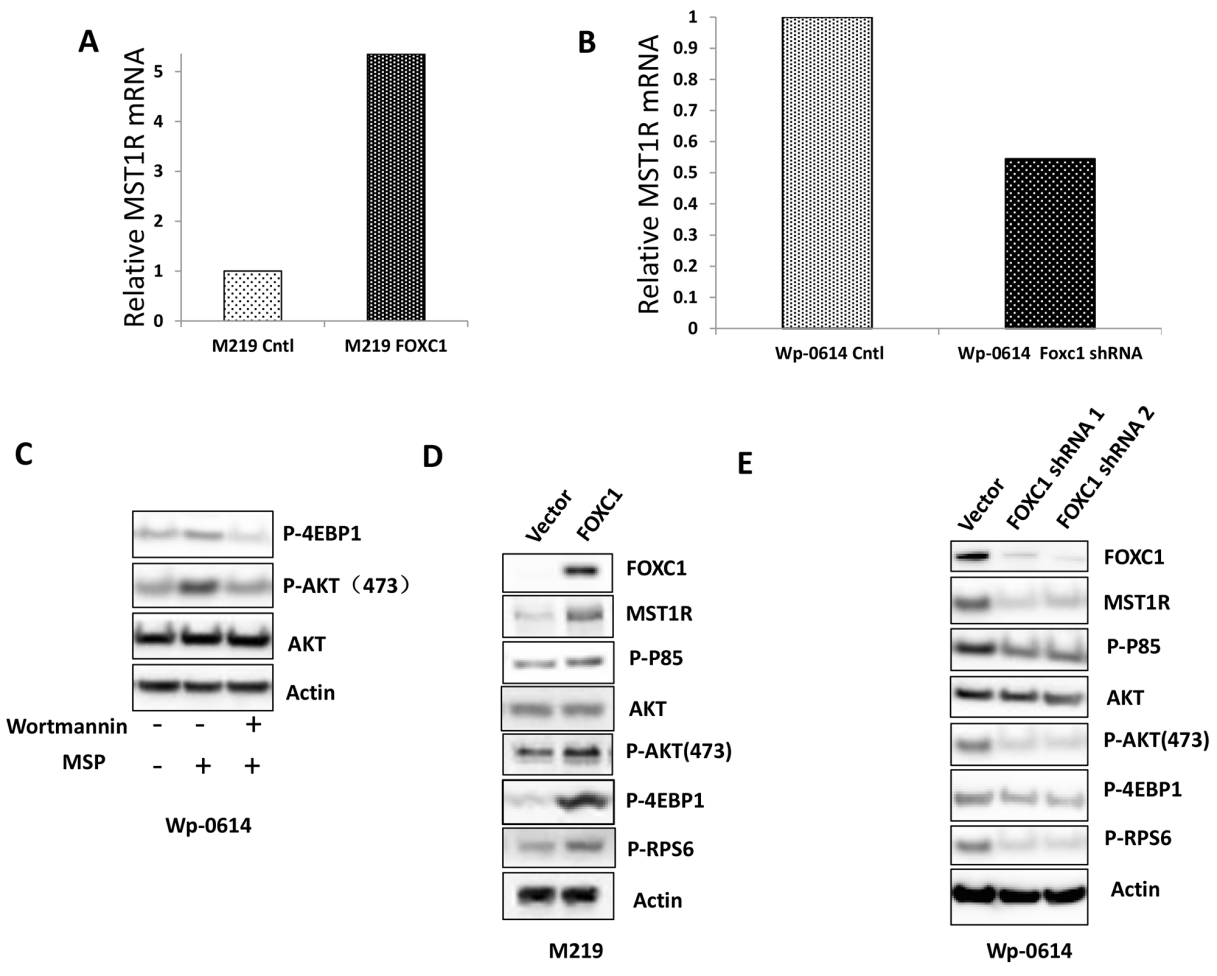
FOXC1 activated MST1R/PI3K/AKT pathway **A.** RNA-Seq data showed that FOXC1 overexpression induced the expression of MST1R. **B.** RNA-Seq data showed that knockdown of FOXC1 reduced the expression of MST1R. **C.** Treatment of MSP increased the expression of p-AKT and p-4EBP1 and the change can be blocked by Wortmannin. **D.** FOXC1 overexpression induced the MST1R expression and activated MST1R/PI3K/AKT pathway. **E.** Knockdown of FOXC1 reduced the MST1R expression and inhibited MST1R/PI3K/AKT pathway.

Our results showed FOXC1 promotes melanoma cell functions *in vitro*, such as growth, migration, invasion, colony formation et al. To check molecular mechanism of FOXC1 function in melanoma, RNA-Seq was done using mRNA from M219 control and M219 FOXC1, M15 control and M15 FOXC1, Wp-0614 control and Wp-0614 FOXC1 shRNA, M14 control and M14 FOXC1 shRNA. It was shown that MST1R (Macrophage Stimulating 1 Receptor) in M219 FOXC1 and M15 FOXC1 was increased compared to M219 control and M15 control, respectively while MST1R in Wp-0614 FOXC1 shRNA and M14 FOXC1 shRNA was reduced compared to Wp-0614 control and M14 control, respectively (Figure 5A and 5B, Supplementary Figure S3A and S3B).

AKT is a common downstream target of MST1R. To examine if AKT was activated when MST1R was increased in cells which have high FOXC1 expression, M219 FOXC1 cells were treated with the PI3K inhibitor Wortmannin (5μM). High expression of MST1R induced by FOXC1 activated AKT and the activation can be blocked by PI3K inhibitor Wortmannin (Figure 5C). To further confirm activation of the MST1R/PI3K/AKT pathway, phosphorylation of p85, phosphorylation of AKT, AKT, downstream targets (phosphorylation of 4EBP1 and RPS6) of the MST1R/PI3K/AKT pathway were assessed. Expression levels of phosphorylated-p85(Tyr458), p-AKT(473) and phosphorylated 4E-BP1 and RPS6 in M219 FOXC1, M15 FOXC1 and Wp-0614 control were higher than that in M219 control, M15 control and Wp-0614 FOXC1 shRNA, respectively (Figure 5D and 5E, Supplementary Figure S4). Taken together, these results suggested that the MST1R/PI3K/AKT pathway was activated in FOXC1 overexpression melanoma cells.

### FOXC1 exerted its function by activating MST1R/PI3K/AKT pathway

**Figure 6:**
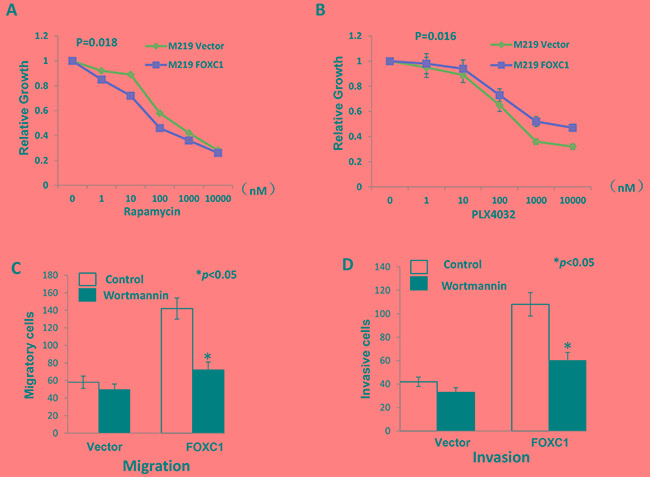
FOXC1 exerted its function by activating MST1R/PI3K/AKT pathway **A.** Rapamycin inhibits the mTOR, which is important component of PI3K/AKT pathway. M219 FOXC1 cells were more sensitive to Rapamycin than M219 control. **B.** PLX4032 is the BRAF inhibitor. M219 FOXC1 cells were more resistant to PLX4032 than M219 control. **C.** Migration of M219 FOXC1 cells were significantly reduced by Wortmannin compared to M219 control cells. **D.** Invasion of M219 FOXC1 cells were significantly reduced by Wortmannin compared to M219 control cells. Error bars, s.d. (*p <0.05).

To investigate if FOXC1 changes sensitivity of melanoma cells to drugs, M219 control and M219 FOXC1 cells were treated by Rapamycin (mTOR inhibitor) or PLX4032 (BRAF inhibitor). It was shown in Figure 6A that M219 FOXC1 was more sensitive to Rapamycin than M219 control. On the contrary, M219 FOXC1 was more resistant to PLX4032 than M219 control (Figure 6B). To test if that FOXC1 affects migration and invasion via PI3K/AKT pathway, M219 control and M219 FOXC1 cells were treated by Wortmannin and cell migration and invasion were analyzed before and after treatment. As shown in Figure 6C and D, migration and invasion of M219 FOXC1 cells were significantly reduced by Wortmannin compared to M219 control cells. MST1R expression was knockdowned by MST1R siRNAs in FOXC1 overexpression melanoma cells and proliferation, migration and invasion of cells were significantly reduced (Supplementary Figure S5 and S6). The similar results were found in M15 control and M15 FOXC1 (Supplementary Figure S7, S8 and S9). In summary, these results suggest that FOXC1 exerted its function by activating MST1R /PI3K/AKT pathway.

### FOXC1 protein expression in melanoma tissues and arrays

**Figure 7:**
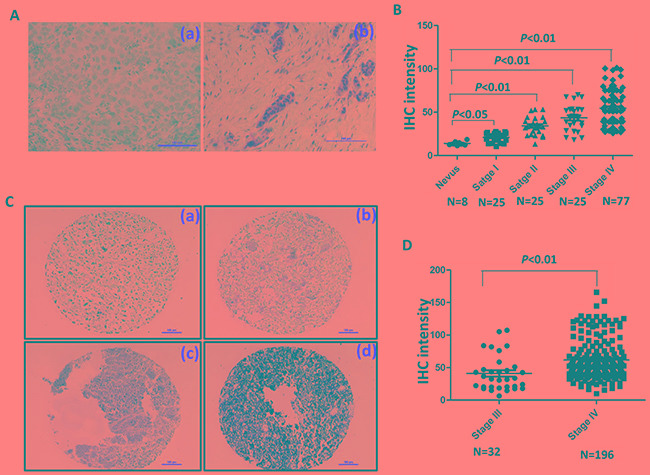
FOXC1 expression is associated with progress of melanoma **A.** Representative IHC pictures of FOXC1 in melanoma tissues. (a) Negative control. (b) FOXC1 expression. **B.** FOXC1 expression was increased as progress of melanoma. FOXC1 expression was significantly lower in nevus than cutaneous melanoma at different stages. **C.** Representative IHC photographs of FOXC1 expression in TMA (Stage III N=32; Stage IV=196). (a) Negative. (b) Weak. (c) Middle. (d) Strong. **D.** There is higher FOXC1 expression in TMA of stage IV (N=196) of than that in TMA of stage III (N=32). Error bars, s.d. (*p <0.05, **p<0.01).

To examine expression of FOXC1 protein in melanoma tissues of different AJCC stages, IHC was performed in our well clinically annotated melanoma PE and TMA. Representative photographs were shown in Figure 7A. It was shown in Figure 7B that FOXC1 expression was increased as progress of melanoma. Representative photographs of TMA IHC were shown in Figure 7C. There was higher FOXC1 expression in TMA of stage IV than that in TMA of stage III (Figure 7D). Altogether, FOXC1 expression was related with progression of melanoma.

### FOXC1 expression is associated with prognosis of melanoma

**Figure 8:**
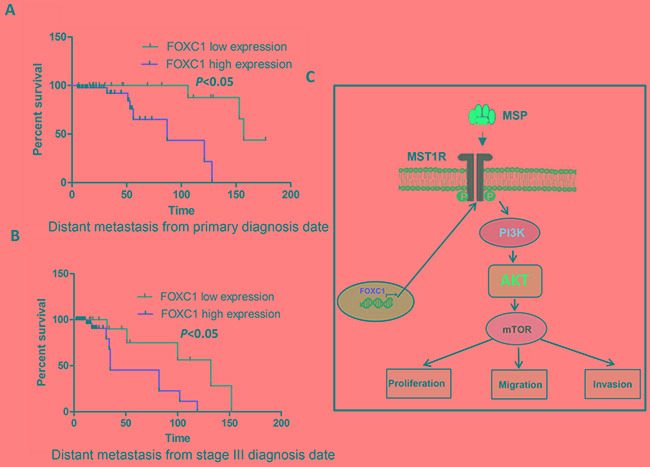
FOXC1 expression is associated with prognosis of melanoma **A.** There is significantly lower distant metastasis free survival rate among patients with high FOXC1 expression than patients with low FOXC1 expression from primary diagnosis date. **B.** There is significantly lower distant metastasis free survival rate among patients with high FOXC1 expression than patients with low FOXC1 expression from stage III diagnosis date. **C.** Schematic model for activating MST1R /PI3K/AKT pathway by FOXC1 overexpression and functional roles of FOXC1 in melanoma cells. *p <0.05.

To assess the clinical significance of FOXC1 in melanoma, Kaplan-Meier analyses were performed. It was shown in Figure 8A and 8B that there was lower distant metastasis free survival rate among patients with high FOXC1 expression than patients with low FOXC1 expression whether calculating from primary diagnosis date or from stage III diagnosis date. These findings suggest that FOXC1 plays a major role in the progression of melanoma.

## DISCUSSION

Many studies showed that FOXC1 was related with breast cancer, liver cancer, lung cancer, stomach cancer and pancreas cancer. Melanoma is the most aggressive skin cancer [20]. However, the mechanism is still unclear. Up to now, there is no FOXC1 study in melanoma. This is the first FOXC1 study on melanoma. In the current study, we showed that FOXC1 was highly expressed in the melanoma cell lines and tissues, that the methylation level of FOXC1 in melanoma is low. FOXC1expression was associated with methylation of FOXC1 gene. FOXC1 promotes cell proliferation, migration and invasion, colony formation and growth in 3D matrigel. Our study also found that FOXC1 induced expression of MST1R and exerted function by MST1R/PI3K/AKT pathway, and that FOXC1 expression was associated with progress and prognosis of melanoma.

Our study firstly showed that alteration of FOXC1 was amplified in melanoma and even more high than most kind cancers (Figure 1A) and that FOXC1 was overexpressed in melanoma cells and tissues. Overexpression of FOXC1 increased tumorigenesis function of melanoma cells while knockdown of FOXC1 reduced tumorigenesis function of melanoma cells. Overexpression of FOXC1 induced expression of P65 and phosph-P65. P65 activation has been found to be correlated with cancer development. Activation of P65 contributes to growth and aggressiveness of papillary thyroid carcinoma [21], migration and invasion of breast cancer cells [22]. P65 expression was also found to be correlated with outcome, such as overall survival (OS), progression-free survival (PFS), and metastasis-free survival (DMFS) in head and neck squamous cell carcinoma [23]. Increase of P65 and phosph-P65 expression may promote proliferation, migration and invasion of FOXC1 overexpression melanoma cells. All in all, these results were similar to findings in previous studies, which further confirmed that FOXC1 promotes development of cancer.

Previous study reported that *foxc1* was hypomethylated and FOXC1 was highly expressed in CD^44+^ breast cancer cells [24]. We analyzed TCGA data and found that methylation level of *foxc1* was low in melanoma. That is the first report about methylation of FOXC1 gene in melanoma. Our results also showed that there is a big CpG island (752 bp) in *foxc1* promoter and FOXC1 expression was associated methylation of FOXC1 gene. Our study further confirmed that there is hypomethylation of *foxc1* in cancers.

PI3K/AKT pathway was closely associated with melanoma. Increased oxidative stress in melanoma cells inhibited PI3K/AKT/mTOR pathway through disruption of mTORC1 formation and phosphorylation of downstream targets p70S6K, 4EBP1 and rpS6 [25]. Both increased DTX3L (Deltex-3-like) level in melanoma cells and DTX3L-mediated regulation of invasion and metastasis in melanoma through FAK/PI3K/AKT but not MEK/ERK signaling was demonstrated [26]. RICTOR (Rapamycin-insensitive companion of mammalian target of rapamycin) plays a central role in PI3K pathway negative feedback in melanocytes and that its deregulation could be involved in melanoma development [27]. Our RNA-Seq data showed overexpression of FOXC1 induced MST1R expression while knockdown of FOXC1 reduced MSTR1 expression. MST1R is also named the receptor tyrosine kinase (RTK) recepteur d'origine Nantais (RON). MST1R is a member of the c-Met family of scatter factor receptors [28, 29]. C-met was highly expressed in melanoma [30]. The macrophage stimulating protein (MSP) is the only known ligand of MST1R. After binding to MSP, RON promotes activation of the PI3K/AKT, MAPK and b-catenin pathways, among others [28, 31]. MSP can increase p-AKT, downstream target p-4EBP1 and the activation can be blocked by Wortmannin in M219 FOXC1 cells. Our further study showed FOXC1 activated MST1R/PI3K/AKT pathway. Oncogene addiction is a common phenomenon in cancers, especially in drug-resistant cancer cells [32–34]. M219 FOXC1 cells were found to be more sensitive to Rapamycin than M219 control while be more resistant to PLX4032 than M219 control. This may be explained by activation of PI3K/AKT pathway by FOXC1. Survival of M219 FOXC1 cells were depended on activation of MST1R/PI3K/AKT pathway and had oncogene addiction, so these cells were more sensitive to Rapamycin. M219 cells which have BRAF mutation were sensitive to PLX4032. Although MAPK/MEK/ERK pathway was blocked by PLX4032, M219 FOXC1 cells can survive via MST1R/PI3K/AKT pathway. This may explain why M219 FOXC1 cells are more resistant to PLX4032 than M219 control and further confirmed previous similar results that Ron receptor tyrosine kinase activation confers resistance to tamoxifen in breast cancer cells [35]. Migration and invasion of M219 FOXC1 cells were significantly reduced by Wortmannin compared to M219 control cells. All these results suggested that FOXC1 activated MRT1R /PI3K/AKT pathway and exerted function via the pathway in melanoma (Figure 8C).

FOXC1 was not only overexpressed in melanoma cell lines, but also overexpressed in melanoma tissues. FOXC1 expression was increased as progress of melanoma. Patients with high FOXC1 expression has lower distant metastasis free survival rate than patients with low FOXC1fromprimary diagnosis date or from stage III diagnosis date. FOXC1 was associated with progress and prognosis of melanoma. FOXC1 may be used as a biomarker for diagnosis or predicting prognosis in melanoma.

In conclusion, our results show that there is high FOXC1 expression in melanoma and that FOXC1 expression was associated with methylation level of FOXC1 gene. Our results also confirmed that FOXC1 activates MST1R/PI3K/AKT pathway and exert function via the pathway. FOXC1 was closely correlated with progress and prognosis of melanoma and played an important role in melanoma. All results suggest that FOXC1 may be used as a theranostic biomarker to treat melanoma and predict the progression of cutaneous melanoma.

## MATERIALS AND METHODS

### Cell culture

We assessed expression of FOXC1 in 10 well-established, early-passaged cutaneous melanoma metastasis cell lines: M14, M15, M24, M101, M204, M219, FD, Wm266-4, DP and Wp-0614 established from AJCC stage III and IV melanoma patients who received surgery at JWCI and normal melanocyte purchased from ATCC (Manassas, VA). Cells were cultured in RPMI 1640 medium supplemented with 10% fetal calf serum, 100U/ml penicillin, and 100 μg/ml streptomycin at 37 °C humidified incubator containing 5%, and were used at early passages. For demethylation assay studies, cultured cells were treated with 2μmol/L 5-aza-2-deoxycytidine (Sigma-Aldrich, St.Louis, MO) dissolved in dimethyl sulfoxide (DMSO) (Sigma-Aldrich, St.Louis, MO) for 72 hours with media changed every 24 hours as previously described [36]. Control cells were treated with DMSO under the same culture conditions.

### Tumor specimens

Approval for the use of human tissues was obtained from the joint IRB of John Wayne Cancer Institute and Providence Saint John's Health Center. Analysis was conducted on paraffin-embedded archival tissue (PEAT) specimens of cutaneous melanoma diagnosed at Providence Saint John's Health Center. Patients were staged using the current AJCC staging system for cutaneous melanoma (AJCC staging manual 7th edition 2010).

### Tissue microarrays

Tissue Microarrays (TMAs) were developed and were clinically well-annotated with > 5yr follow-up, as previously described [37, 38]. AJCC stage III and IV melanoma TMAs included 268 distant organ metastases and 39 autologous stage III lymph node metastases, as well as 29 cancer free normal tissues from each respective organ as controls.

### Stable transfection

M14, M15, M219 and Wp-0614 cells were plated in 60 dishes at 80% confluence before transfection 24h. FOXC1-myc-flag plasmid (Origene, Rockville, MD) and FOXC1 shRNAs (Sigma-Aldrich, St.Louis, MO) were stably transfected into M15, M219, M14 and Wp-0614 using Lipofectamine™ 3000 transfection reagent (Invitrogen, Grand Island, NY) for 24 h. The M219, M15 cells which have high FOXC1 overexpression were then screened under 0.8 mg/ml G418 (Invitrogen, Grand Island, NY) for 3 weeks while Wp-0614, M14 cells which have low FOXC1 expression were selected in 5 μg/ml puromycin. Cell clones which have overexpressing FOXC1 were suncloned as M219 FOXC1 and M15 FOXC1 while cell clones which have low FOXC1 expression were suncloned as Wp-0614 FOXC shRNA and M14 FOXC shRNA. Expression of FOXC1 was verified by western blot analysis with anti-FOXC1 antibody (Santa Cruz Biotechnology, Santa Cruz, CA), anti-myc antibody (EMD Millipore, San Diego, CA) and anti-flag antibody (Origene, Rockville, MD). FOXC1 shRNA sequences were seen in Supplementary Table S1.

For siRNA transfection, double-stranded, siRNAs (21-mer) targeting MST1R were designed and synthesized by genePharma company (www.genepharm.com). The corresponding target MST1R mRNA sequences for the siRNAs were as follows: MSR1R siRNA1: TCGCGACTTTGACGTGAAGTACG; MSR1R siRNA2: GGCGACAGAAATGAGAGTGCTGT. There siRNAs were transfected into M219 FOXC1 cell lines by Lipofectamine® RNAiMAX transfection reagent (Invitrogen, Grand Island, NY) according to the manual. Two days after transfection, cells were used to proliferation, migration and invasion assay.

### Cell growth

Cell proliferation and viability were assessed by using 3-(4,5-dimethylthiazol-2-yl)-2,5-diphenyltetrazolium (MTT) obtained from Sigma-Aldrich (St. Louis, MO) in RPMI 1640 supplemented with 10% heat-inactivated FBS at 37^o^C in a humidified incubator with 5% CO_2_. 3D cell culture was performed using Matrigel matrix (BD Biosciences, San Jose, CA) in a 96-well microplate according to the manufacturer's instructions.

As for drug sensitivity experiments, M219 control, M15 control, M15 FOXC1 and M219 FOXC1 cells were planted in 96 well plates and were treated by Rapamycin or PLX4032.

### Cell migration and invasion assays

Migration and invasion assays were carried out according to the manufacturer's instructions (BD Biosciences, San Jose, CA). Cells (2.5x10^4^) were briefly placed in transwell migration (BD, Catalog Number: 354578) and invasion chambers (BD, Catalog Number: 354480). After 24 hours, cells on the lower surface were fixed with methanol and stained with 2% crystal violet solution. The membrane was then mounted onto a microscope slide and the migrating cells were counted in five different areas using a light microscope. The experiments were performed three times in triplicate.

### Soft agar colony assay formation

A soft agar colony formation assay was done using six-well plates. Each well contained 2 mL of 0.7% agar in complete medium as the bottom layer, 1 mL of 0.35% agar in complete medium and 3,000 cells as the feeder layer, and 1.5 mL complete medium as the top layer. Cultures were maintained under standard culture conditions for 3–4 weeks. The colonies were stained with MTT solution at the termination of culture.

### Real-time reverse transcription PCR

Total RNA was isolated from 11 cell lines cells using the RNeasy mini kit (Qiagen, Hilden, Germany), with on-column DNase treatment to remove contaminating genomic DNA. Real time reverse transcription-PCR (RT-PCR) was done as in reference [39]. The primers (Integrated DNA Technologies, Inc., Coralville, Iowa) for RT-PCR were listed in Supplementary Table S2.

### Immunoblot analysis

Whole cell extracts were prepared from M219 control and M219 FOXC1, M15 control and M15 FOXC1, Wp-0614 control and Wp-0614 FOXC1 shRNA cells. Western blot assays were done as previously described (12). Immunoblotting was done with polyclonal antibodies against FOXC1, Cyclin D1 (1:200; Santa Cruz Biotechnology, Santa Cruz, CA), monoclonal antibody against P65 (1:200, Santa Cruz Biotechnology, Santa Cruz, CA), monoclonal antibody against MST1R (Thermo Scientific, Rockford, lL), polyclonal antibodies against p-p85, p-AKT, AKT, p-RPS6 (1:1000, 1:500, Cell Signaling, Danvers, MA), monoclonal antibody against p-4EBP1 (1:1000, 1:500, Cell Signaling, Danvers, MA). Anti-β actin antibody (Sigma-Aldrich, St.Louis, MO) was used at a 1:10000 dilution. Incubation with primary antibodies overnight was followed by incubation with secondary antibody (1:4000; Anti-mouse IgG NA931V, Anti-rabbit IgG NA934V GE Biosciences and 1:4000, Anti-goat IgG, Biotechnology, Santa Cruz, CA). Detection was carried out using the Pierce SuperSignal West Pico chemiluminescent substrate (Thermo Scientific, Rockford, lL) followed by scanning using a Fluorchem 5500 chemiluminescence imager (Alpha Innotech Corp, San Leandro, CA).

### Methylation-specific PCR (MS-PCR)

The MS-PCR assays were performed in triplicate and designed to amplify bisulfite-converted methylated DNA target sequences as previously described[40]. The methylation-specific primers and unmethylated-specific primers are listed in Supplementary Table S3. For the MS-PCR, 2 μg of DNA was used for each reaction.

### Immunohistochemistry

Five-micrometer paraffin-embedded tissue sections were deparaffinized and rehydrated, antigens were retrieved, and IHC was performed using an optimized protocol. Slides were deparaffinized, rehydrated and washed in 1X PBS. Antigen retrieval was performed with 1X citrate buffer (Sigma-Aldrich, St.Louis, MO) at 100°C for 10 min and then incubated in H_2_O_2_ (Sigma-Aldrich, St.Louis, MO) at room temperature to block endogenous peroxidase. Separate slides were incubated in primary rabbit Anti-FOXC1 antibody (aa250-300) IHC-plus™ LS-B1800 (1:250 dilution; Seattle, WA ) overnight in a 4°C humid chamber followed by 1 hr incubation with secondary biotinylated link Ab. The reaction for FOXC1 was developed using a labeled streptavidin biotin (LSAB) method (LSAB+ Kit; Dako, Carpinteria, CA) and visualized using VIP Substrate Kit (Vector Laboratories, Burlingame, CA). Specificity of the immunostaining was determined by the inclusion of isotype-specific IgG (Santa Cruz Biotechnology, Santa Cruz, CA) as negative controls. The sections were counterstained with hematoxylin (Sigma-Aldrich). Photographs of each IHC-stained section were taken for analysis using a Nikon Eclipse Ti microscope and NIS elements software (Nikon, Melville, NY). Staining density was determined by Image J software (http://rsbweb.nih.gov/ij/). After adjustment for background on each selected field, the density of the individual breast cancer specimen was quantified and given a numerical value from 0–255. Melanoma specimens were tested in duplicate, and the average of the two staining intensity values was used for statistical analysis.

### RNA deep sequencing

Samples of high quality RNA (RIN ≥ 8.0) were used to create mRNA libraries using the Illumina TruSeq RNA Sample Preparation Kit v2. The mRNA libraries were then sequenced on the Illumina HiSeq 2500 high-throughput mode using TruSeq® SBS v3–HS 200 cycle kit according to standard procedures generating over 35 million 100-base pair paired-end reads per sample(Illumina, San Diego, CA). Base calling and demultiplexing were processed using CASAVA v1.8 (Illumina, San Diego, CA), alignment was performed using TopHat2 and expression values were generated using Cufflinks [41, 42]. The experiment wasn't repeated.

### Statistical analysis

The results are given as mean ± SD of samples measured in triplicate. Each experiment was repeated three times, unless otherwise indicated. Student's t-test was used to calculate differences between the various study groups. Results of IHC intensity in different groups were analyzed by ANOVA. The difference was considered statistically significant at *p* <0.05.

## SUPPLEMENTARY MATERIALS FIGURES AND TABLES


