# Correction: FOXC1 promotes melanoma by activating MST1R/PI3K/AKT pathway and is associated with poor prognosis in melanoma

**DOI:** 10.18632/oncotarget.28590

**Published:** 2024-06-13

**Authors:** Jinhua Wang, Li Li, Shiwei Liu, Ying Zhao, Lin Wang, Guanhua Du

**Affiliations:** ^1^The State Key Laboratory of Bioactive Substance and Function of Natural Medicines, Beijing Key Laboratory of Drug Target Research and Drug Screen, Institute of Materia Medica, Chinese Academy of Medical Science and Peking Union Medical College, Beijing 100050, China; ^2^Department of Endocrinology, Shanxi DAYI Hospital, Shanxi Medical University, Taiyuan, Shanxi 030002, China; ^3^Department of Molecular Oncology, John Wayne Cancer Institute (JWCI) at Providence Saint John’s Health Center, Santa Monica 90404, CA, USA

**This article has been corrected:** In Figure 2C, the Wp-0614 Cntl migration image (lower left panel) is incorrect; the wrong picture was mistakenly selected during figure preparation. The corrected Figure 2C, obtained using the original data, is shown below. The authors declare that these corrections do not change the results or conclusions of this paper.

Original article: Oncotarget. 2016; 7:84375–84387. 84375-84387. https://doi.org/10.18632/oncotarget.11224

**Figure 2:**
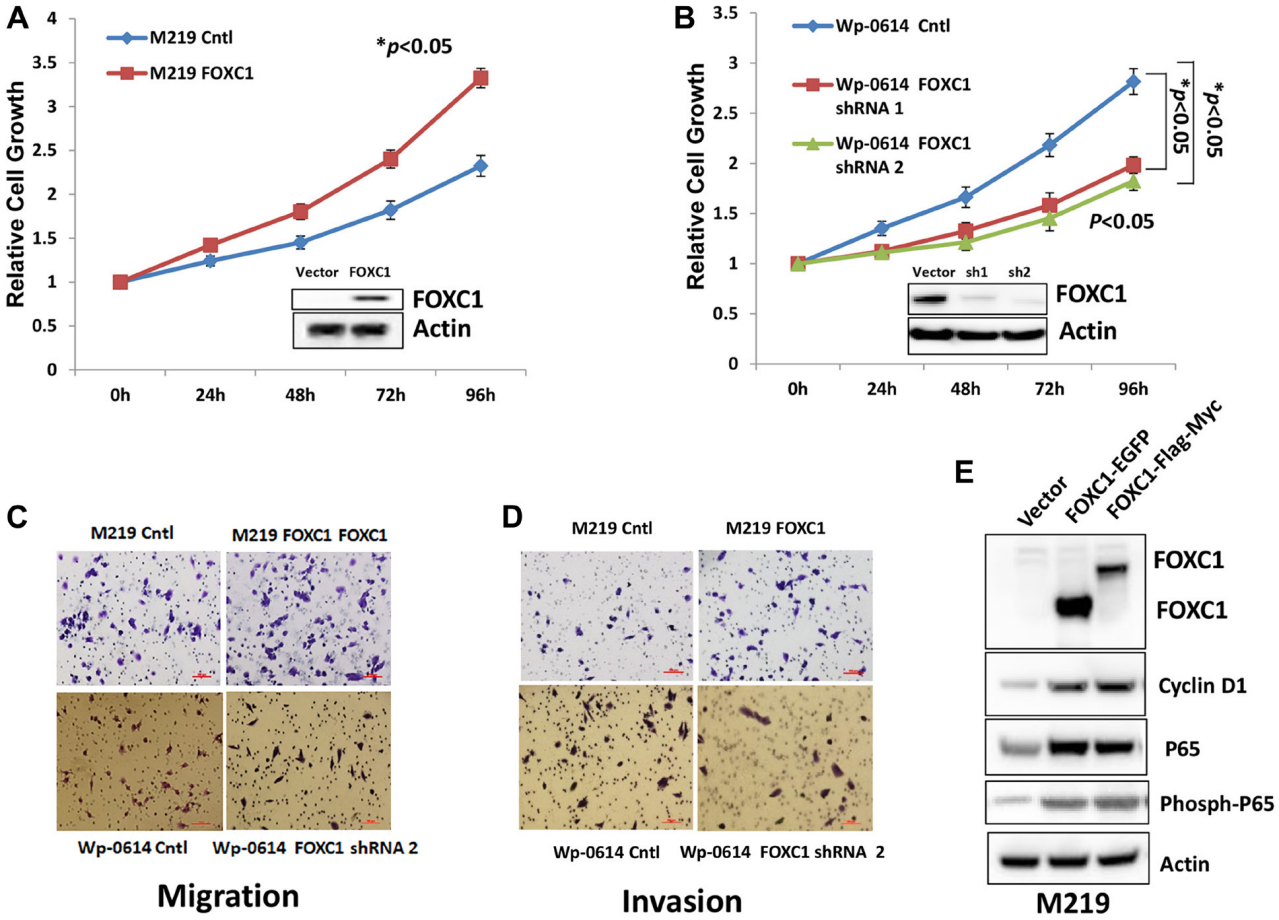
FOXC1 promotes proliferation, migration and invasion of melanoma cells. (**A**) The growth of M219 FOXC1 is higher than that of M219 control. (**B**) The growth of Wp-0614 FOXC1 shRNA is lower than that of Wp-0614 control. (**C**) FOXC1 overexpression increased cell migration while knockdown of FOXC1 reduced cell migration. (**D**) FOXC1 overexpression increased cell invasion while knockdown of FOXC1 reduced cell invasion. (**E**) FOXC1 overexpression induced expression of Cyclin D1, P65 and phosph-P65, which are related to growth, migration and invasion of melanoma cells. Error bars, s.d. (^*^ *p* < 0.05).

