# Supplementary material for: FOXC1 promotes melanoma by activating MST1R/PI3K/AKT pathway and is associated with poor prognosis in melanoma
Source: Oncotarget. 2016 Aug 11;7(51):84375–87. doi: 10.18632/oncotarget.11224 (PMC5356666; doi:10.18632/oncotarget.11224)
Supplement: Supplementary file 1 [file oncotarget-07-84375-s001.pdf]

FOXC1 promotes melanoma by activating MST1R/PI3K/AKT pathway and is associated with poor prognosis in melanoma

SUPPLEMENTARY FIGURES AND TABLES

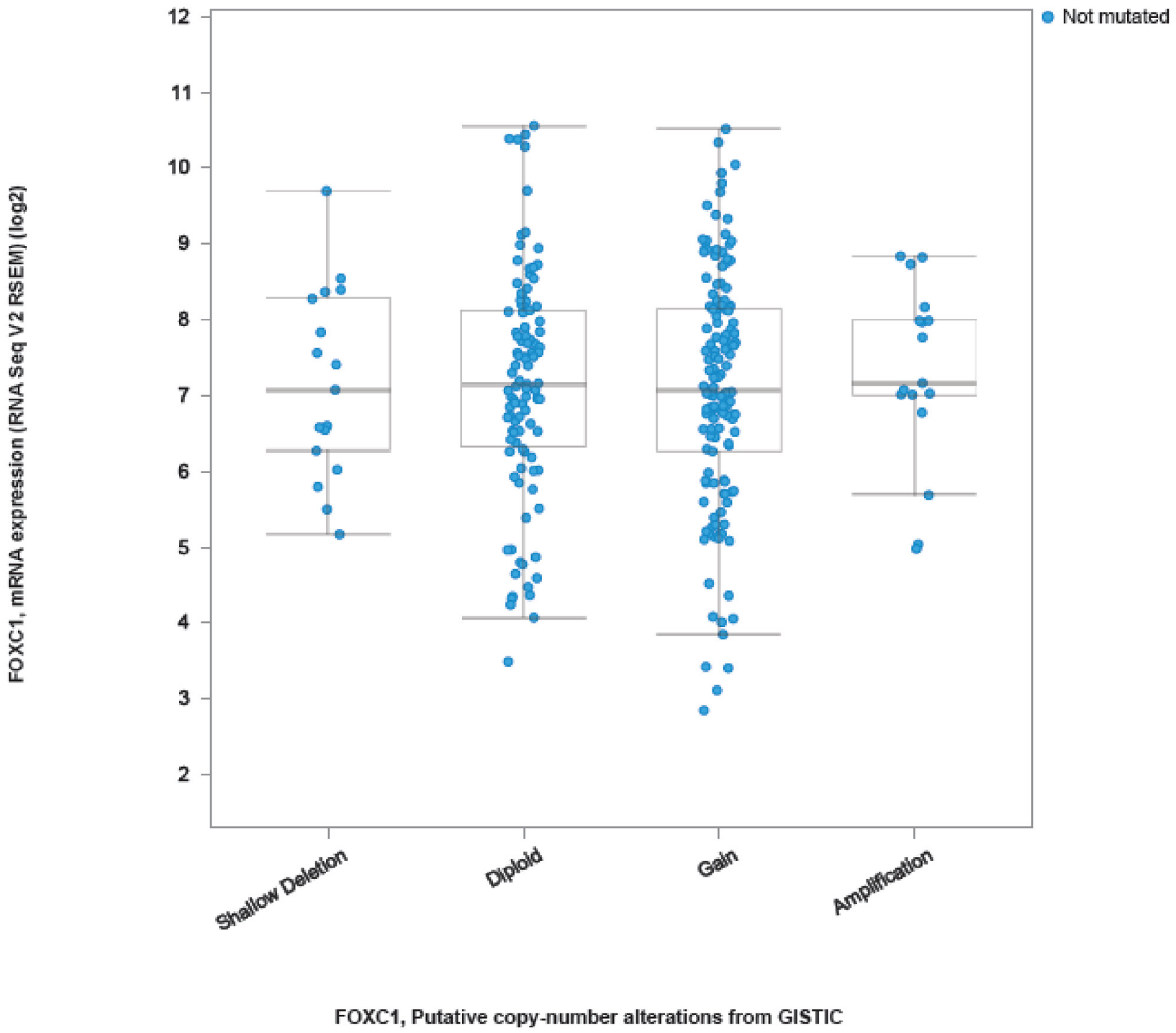

Supplementary Figure S1: FOXC1 amplification and FOXC1 mRNA expression. TAGA data shows that FOXC1 is amplified in 6-7%. FOXC1 mRNA was investigated in same TCGA dataset using in silicon assay. Relation between FOXC1 mRNA and FOXC1 copy number was showed. There is higher FOXC1 expression in FOXC1 amplification group. X-axis: Copy number; Y-axis: mRNA expression.

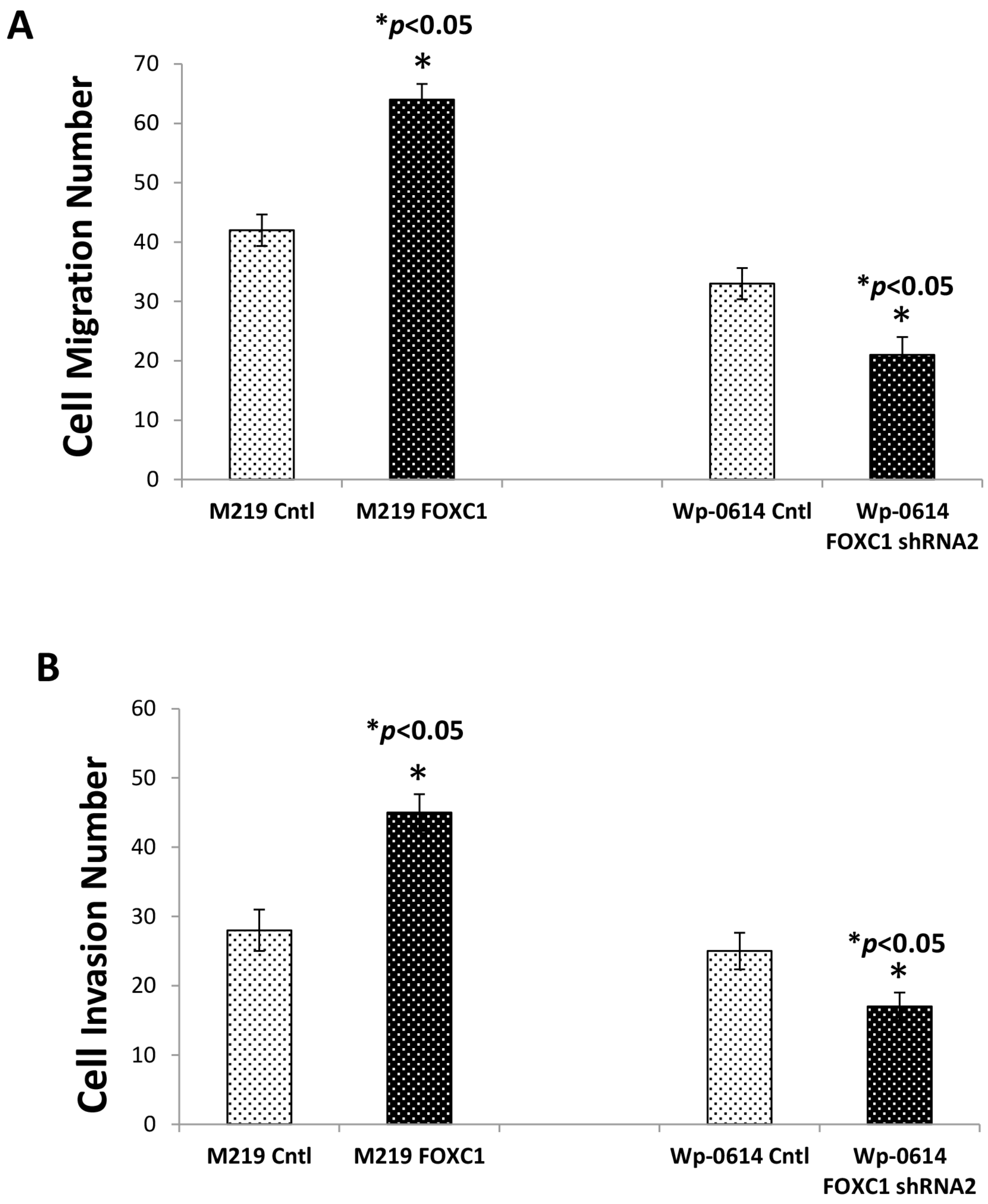

Supplementary Figure S2: Cell migration and invasion of M219 control and M219 FOXC1, Wp-0614 control and Wp-0614 FOXC1 shRNA. A. Quantification of migration (N=3). FOXC1 overexpression increased cell migration and FOXC1 shRNA reduced cell migration. **B.** Quantification of invasion (N=3). FOXC1 overexpression increased cell invasion and FOXC1 shRNA reduced cell invasion. Error bars, s.d. (*$p < 0.05$).

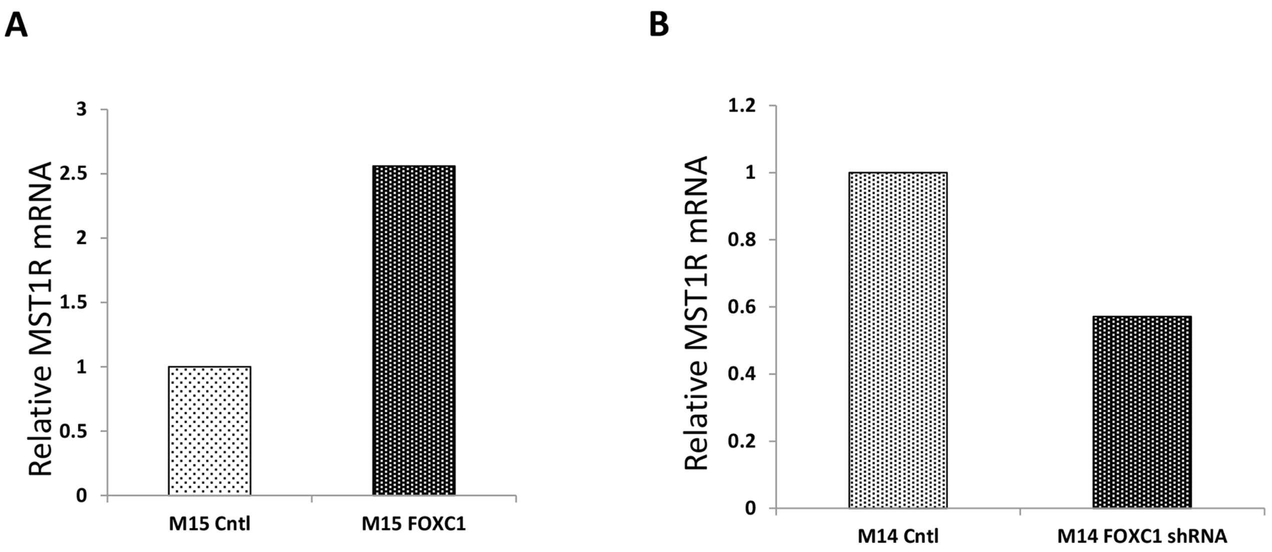

Supplementary Figure S3: FOXC1 induced MST1R expression. RNA-Seq showed that expression of MST1R in FOXC1 M15 was higher than that in M15 control. RNA-Seq showed that expression of MST1R in M14 FOXC1 shRNA was lower than that in M14 control.

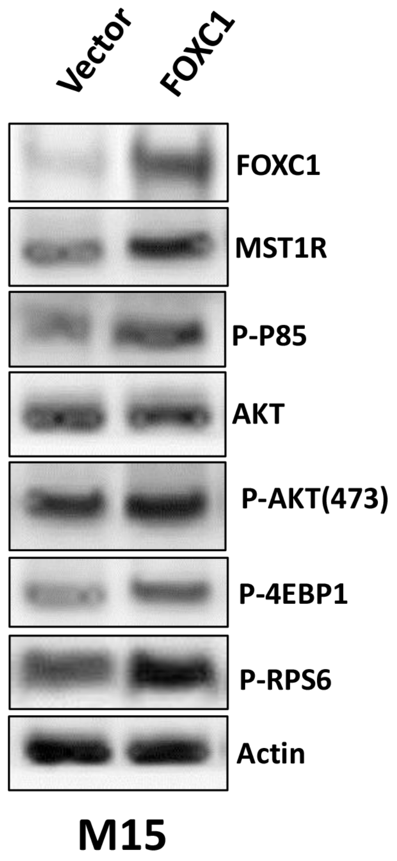

Supplementary Figure S4: FOXC1 activated MST1R/PI3K/AKT pathway. There is low FOXC1 expression in M15 cells. M15 cells were transfected with FOXC1 expression plasmid. Stable cells clones which have high FOXC1 were selected by G418. FOXC1 overexpression induced the MST1R expression and activated MST1R/PI3K/AKT pathway in M15 FOXC1 cells.

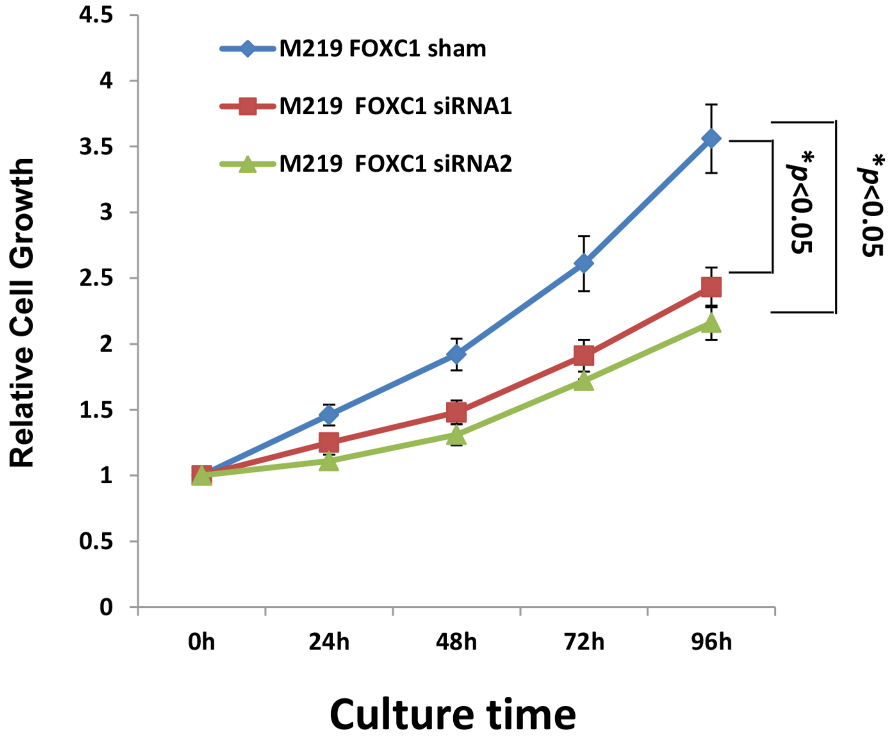

Supplementary Figure S5: FOXC1 siRNA inhibited proliferation of M219 FOXC1 cell. M219 FOXC1 cell were transfected with FOXC1 siRNA using Lipofectamine® RNAiMAX transfection reagent. After transfection 48 hours, cells were used to do proliferation assay. Results showed that FOXC1 siRNA inhibited cell proliferation. Error bars, s.d. (*$p < 0.05$).

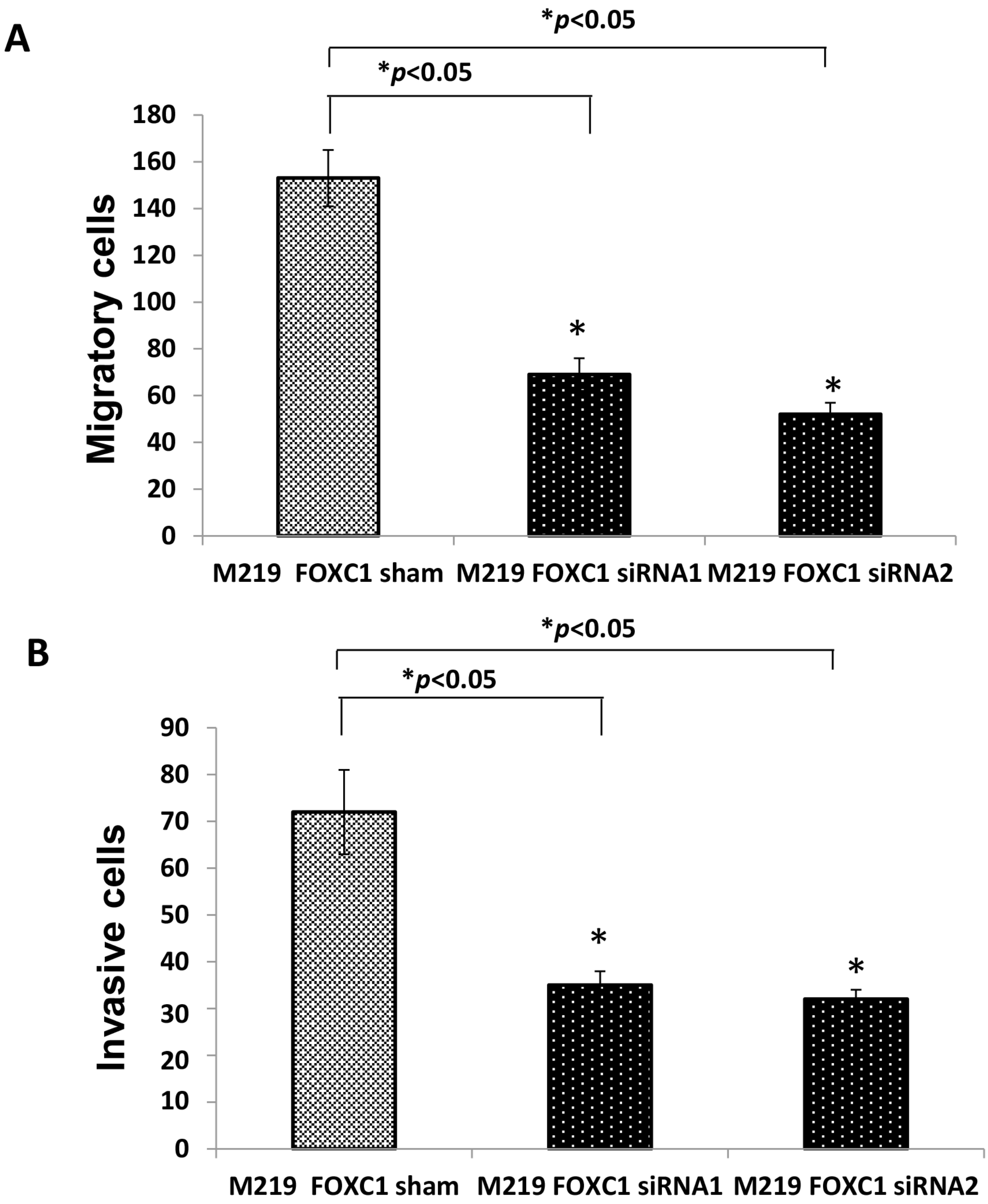

Supplementary Figure S6: FOXC1 siRNA inhibited migration and invasion of M219 FOXC1 cell. M219 FOXC1 cell were transfected with FOXC1 siRNA1 using Lipofectamine® RNAiMAX transfection reagent. After transfection 48 hours, cells were used to do proliferation assay. **A.** FOXC1 siRNA inhibited cell migration. **B.** FOXC1 siRNA inhibited cell invasive. Error bars, s.d. (*$p<0.05$).

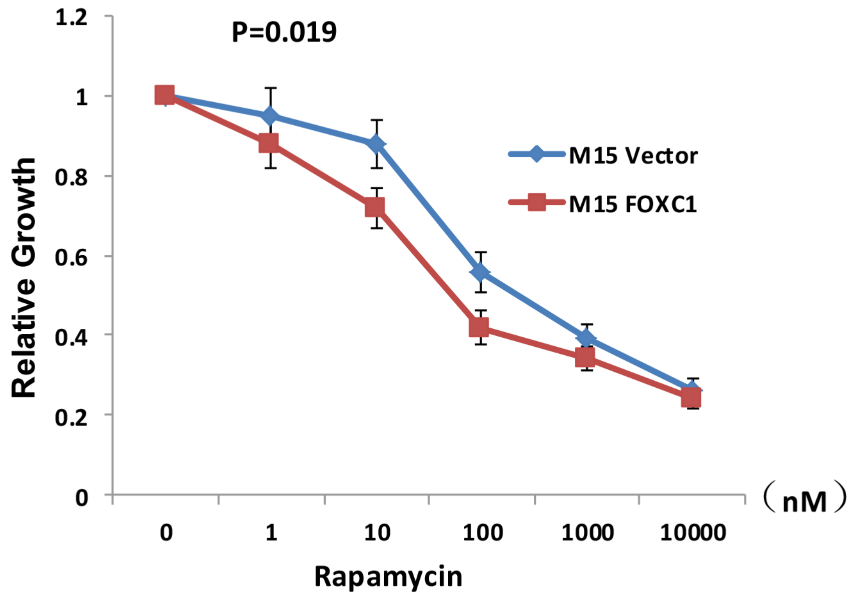

Supplementary Figure S7: FOXC1 expression rendered cells sensitivity to Rapamycin. Rapamycin inhibits the mTOR, which is important component of PI3K/AKT pathway. M15 FOXC1 cells were more sensitive to Rapamycin than M15 control. Error bars, s.d. (**p* <0.05).

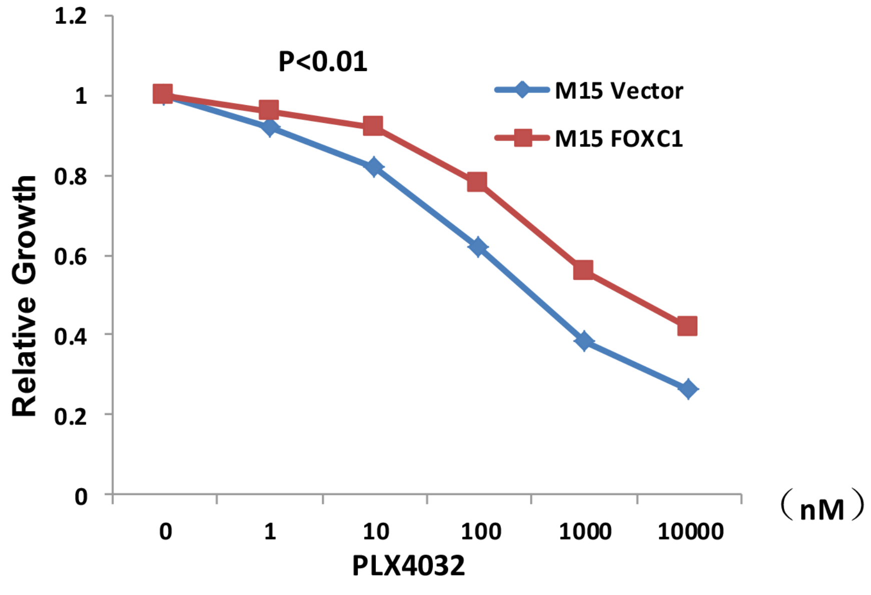

Supplementary Figure S8: FOXC1 expression caused cell resistance to PLX4032. PLX4032 is the BRAF inhibitor and used to treat melanoma. M15 FOXC1 cells were more resistant to PLX4032 than M15 control. Error bars, s.d. (**p* <0.05).

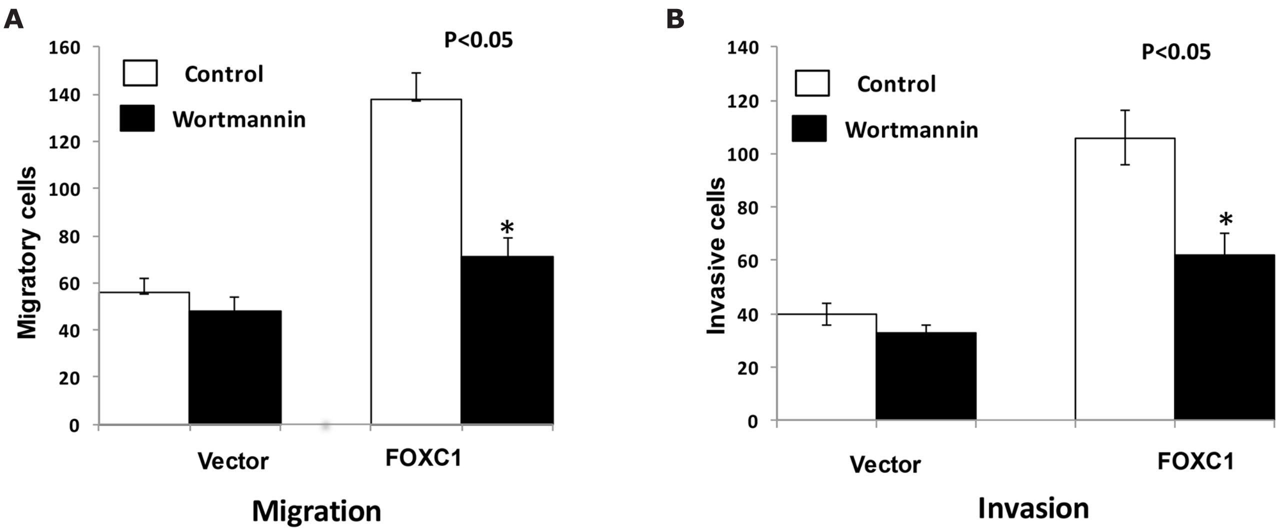

Supplementary Figure S9: FOXC1 promoted migration and invasion of cells. A. Migration of M15 FOXC1 cells were significantly reduced by Wortmannin compared to M15 control cells. **B.** Invasion of M15 FOXC1 cells were significantly reduced by Wortmannin compared to M15 control cells. Error bars, s.d. (**p* <0.05).

Supplementary Table S1: FOXC1 shRNA sequences

The FOXC1 shRNA sequence 1:<br>CCGGGAGCTTTCGTCTACGACTGTACTCGAGTACAGTCGTAGACGAAAGCTCTTTTTG
The FOXC1 shRNA sequence 2:<br>CCGGACTCTCCAGTGAACGGGAATACTCGAGTATTCCCGTTCACTGGAGAGTTTTTTG

Supplementary Table S2: Primers for RT-qPCR assays

Biomarker	Forward	Reverse
FOXC1	CGG TAT CCA GCC AGT CTC TGT AC[FAM]G	GTT CGG CTT TGA GGG TGT GTC
B2-microglobulin	TGTCACAGCCCAAGATAG	CCAGCAAGCAGAATTTGGAA

Supplementary Table S3: Primers for MS-PCR

	Forward	Reverse
M primer	GTTTTTTTAATGTTTTTTTAAGCGG	AACTCTACCCTAAACTACCGACGA
U primer	GTTTTTTTAATGTTTTTTTAAGTGG	AAAAAACTCTACCCTAAACTACCAACA
